# Breviscapine remodels myocardial glucose and lipid metabolism by regulating serotonin to alleviate doxorubicin-induced cardiotoxicity

**DOI:** 10.3389/fphar.2022.930835

**Published:** 2022-09-27

**Authors:** Meng-Jiao Li, Wen-She Sun, Yang Yuan, Yu-Kun Zhang, Qi Lu, Yuan-Zhen Gao, Ting Ye, Dong-Ming Xing

**Affiliations:** ^1^ Cancer Institute of the Affiliated Hospital of Qingdao University and Qingdao Cancer Institute, Qingdao, China; ^2^ School of Basic Medicine, Qingdao University, Qingdao, China; ^3^ School of Life Sciences, Tsinghua University, Beijing, China

**Keywords:** doxorubicin, DIC, breviscapine, serotonin, PINK1/Parkin

## Abstract

**Aims:** The broad-spectrum anticancer drug doxorubicin (Dox) is associated with a high incidence of cardiotoxicity, which severely affects the clinical application of the drug and patients’ quality of life. Here, we assess how Dox modulates myocardial energy and contractile function and this could aid the development of relevant protective drugs.

**Methods:** Mice were subjected to doxorubicin and breviscapine treatment. Cardiac function was analyzed by echocardiography, and Dox-mediated signaling was assessed in isolated cardiomyocytes. The dual cardio-protective and anti-tumor actions of breviscapine were assessed in mouse breast tumor models.

**Results:** We found that Dox disrupts myocardial energy metabolism by decreasing glucose uptake and increasing fatty acid oxidation, leading to a decrease in ATP production rate, an increase in oxygen consumption rate and oxidative stress, and further energy deficits to enhance myocardial fatty acid uptake and drive DIC development. Interestingly, breviscapine increases the efficiency of ATP production and restores myocardial energy homeostasis by modulating the serotonin-glucose-myocardial PI3K/AKT loop, increasing glucose utilization by the heart and reducing lipid oxidation. It enhances mitochondrial autophagy *via* the PINK1/Parkin pathway, eliminates damaged mitochondrial accumulation caused by Dox, reduces the degree of cardiac fibrosis and inflammation, and restores cardiac micro-environmental homeostasis. Importantly, its low inflammation levels reduce myeloid immunosuppressive cell infiltration, and this effect is synergistic with the anti-tumor effect of Dox.

**Conclusion:** Our findings suggest that disruption of the cardiac metabolic network by Dox is an important driver of its cardiotoxicity and that serotonin is an important regulator of myocardial glucose and lipid metabolism. Myocardial energy homeostasis and timely clearance of damaged mitochondria synergistically contribute to the prevention of anthracycline-induced cardiotoxicity and improve the efficiency of tumor treatment.

## Introduction

The anthracycline antibiotic doxorubicin (Dox) is a highly effective anticancer agent for the treatment of solid tumors, leukemias, lymphomas, and breast cancer (Takemura and Fujiwara, 2007). Dox use can lead to a progressive, chronic, life-threatening cardiomyopathy known as Dox-induced cardiomyopathy (DIC) (Levis et al., 2017; Sawicki et al., 2021). Notably, in patients with cancer, the risk of death from drug-related cardiotoxicity exceeds the risk of death from the tumor itself or recurrence (Liu et al., 2017). DIC resulting from cardiotoxicity may occur after the administration of low doses of Dox depending on an individual’s susceptibility (McGowan et al., 2017). Epidemiological studies have shown that metabolic abnormalities, such as obesity, diabetes, and liver disease, lead to an increased risk of DIC and that Dox robustly reduces the absorption of glucose by the heart, suggesting that Dox may affect the development of DIC by disrupting the metabolic microenvironment of the heart. Recently, studies on the potential molecular mechanisms of DIC have focused on red ox homeostasis imbalance, topoisomerase IIβ (Top2b) inhibition of transcription, mitochondrial dysfunction, and Ca^2+^-handling abnormalities (Vejpongsa and Yeh, 2014; Kalyanaraman, 2020; Hulst et al., 2021). The heart consumes the greatest amount of energy in the body, and Dox reshapes cardiac metabolism. However, the alterations that drive the development of DIC are not fully understood. Therapeutic strategies for preventing DIC through the regulation of cardiac metabolic networks have not been established.

Cardiac energy metabolism involves intricate sets of interacting pathways that result in the use of the energy substrate of each class for ATP production or biosynthesis. The most salient feature of the network is the metabolic flexibility demonstrated in response to various stimuli, including developmental changes, nutritional status, chronic pathophysiological conditions, and pharmacological interventions (Murashige et al., 2020). Mitochondria, which are multipurpose organelles that account for one-third of the volume of cardiomyocytes and not only generate more than 95% of the ATP utilized by the heart but also regulate intracellular calcium homeostasis, signal transduction, and cell death, are central to coordinated energy transduction (Kolwicz et al., 2013). Although the heart is capable of utilizing all kinds of energy substrates, including carbohydrates, lipids, amino acids, and ketone bodies, for ATP production, the substrate used affects cardiac efficiency. Elevated fatty acid concentrations and oxidation are associated with decreased glucose oxidation in the context of obesity and diabetes (Randle et al., 1963; Li and Zhang, 2016). Cardiac dysfunction is associated with increased myocardial oxygen consumption, reduced cardiac efficiency, and increased oxidative stress, suggesting that increased fatty acid oxidation (FAO) is detrimental to cardiac function (Lopaschuk et al., 2010; Lopaschuk et al., 2021; Yamamoto and Sano, 2022). One mechanism underlying the adverse effects of high FAO is the decrease in oxygen efficiency and an increase in the levels of fatty acid derivatives, which may further reduce efficiency by uncoupling mitochondria. In fact, the elevation of lipid peroxidation (LP) is observed in DIC, suggesting that high FAO may be a key pathway for cardiac injury. Therefore, we hypothesize that cardiac metabolic remodeling during Dox treatment increases fatty acid uptake and oxidation, resulting in low ATP production efficiency, accompanied by high oxygen consumption and oxidative stress, and that reduced cardiac efficiency, in turn, feeds back to enhanced fatty acid utilization and mitochondrial damage, which in turn accelerates the progression of DIC.

Interestingly, epidemiological studies suggest that regular consumption of flavonoid-rich foods may reduce the risk of many cardiovascular diseases, and many natural flavonoid compounds have been shown to protect heart function (Shabalala et al., 2017; Syahputra et al., 2022). *Erigeron breviscapus*, also known as *Asherpa breviscapine* or Apocynum, is a traditional herb that has been used for over 600 years. Breviscapine is a flavonoid component of *Erigeron breviscapus* that has a wide range of pharmacological effects, such as antioxidant, anti-inflammatory, and antitumor effects (Gao et al., 2017; Wen et al., 2021). Breviscapine injection is the most widely used classical drug for the treatment of cardiovascular and cerebrovascular diseases in China, and it is also an important drug for emergency treatment in Chinese hospitals. Breviscapine has been clinically applied for the treatment of hypertension, cerebral embolism, and cerebrovascular diseases for more than 20 years (Wu et al., 2019). However, there are currently no reports on its protective effect against cardiac injury caused by chemotherapy drugs.

In this study, we explored the potential effects of breviscapine on DIC and its possible molecular mechanisms by establishing an anthracycline-induced myocardial injury model using H9c2 rat cardiomyocytes and C57BL/6 mice. We also systematically elucidated that breviscapine can improve myocardial glucose utilization efficiency through the glucose-regulated branched-serotonin pathway, thus exerting insulin-like functions to achieve high ATP production efficiency and low oxidative stress levels during lower oxygen consumption, and it can activate the classical mitochondrial PINK1/Parkin autophagic pathway to eliminate Dox-induced accumulation of damaged mitochondria, coordinate the regulation of oxidative stress and energy production, and gradually restore the homeostasis of the cardiac microenvironment. It is thought that breviscapine is a potential novel protective agent for doxorubicin-induced cardiotoxicity.

## Materials and methods

All animal studies were approved by and performed according to the guidelines of the University of Qingdao Animal Care and Use Committee.

### Reagents

The following reagents were used: DMEM, FBS, penicillin/streptomycin (Meilunbio, Dalian, China), cTnI Elisa kits (Sangon Biotech, Shanghai, China), TNF-α, IL-1β, and IL-6 Elisa kits (MyBioSource, San Diego, United States), MDA kits, SOD kits, and NADH kits (Solarbio, Beijing, China), CCK-8 kits (Beyotime Biotechnology, Shanghai, China), ROS fluorimetric kits, JC-1 fluorescent dye, protein extraction kits, and BCA kits (Meilunbio, Dalian, China). IL-1 (1:100), PINK1(1:1,000), Parkin (1:1,000), AMPK(1:1,000), Akt (1:1,000), P13K(1:1,000), Tom20 (1:1,000), β-tubulin (1:1,000), mTOR (1:1,000), and an HRP-conjugated GAPDH (1:1,000) antibody were obtained from Abclonal Biotechnology (Wuhan, China). The RIPA lysis buffer and loading buffer were purchased from Meilunbio (Dalian, China). PVDF membranes were obtained from Merck (New Jersey, United States). DMSO was purchased from Macklin (Shanghai, China), and doxorubicin, breviscapine, and dexrazoxane were purchased from Widely (Wuhan, China). The Mdivi-1 inhibitor was purchased from Selleck Chemicals (Houston, United States).

### Cell culture conditions and cell lines

Culture medium and supplements for cell culture were purchased from Gibco-Invitrogen (Carlsbad, CA, United States), and plasticware was purchased from Corning (Corning, NY, United States). H9c2 embryonic rat heart-derived cardiomyoblast cells (ATCC, CRL-1446) were cultured in DMEM containing 10% fetal bovine serum, 100 units/ml penicillin G sodium, and 100 μg/ml streptomycin sulfate (37°C, 5% CO_2_). MCF-7 human metastatic breast cancer cells (iCell, iCell-h129) were cultured in 1,640 media supplemented with 10% fetal bovine serum, 100 units/ml penicillin G sodium, and 100 μg/ml streptomycin sulfate. 4T1 mouse breast cancer cells (ATCC, FS-0158) were cultured in DMEM medium supplemented with 10% fetal bovine serum, 100 units/ml penicillin G sodium, and 100 μg/ml streptomycin sulfate.

The cells were passaged regularly when they reached 80–90% confluence and seeded in 96-well plates (1 × 10^4^ cells/well). H9c2 cells were exposed to control, 5 μM Dox (Guo et al., 2013), 5 μM Dox+20 μM Dexra (Sangweni et al., 2020), or 5 μM Dox+200 μM breviscapine (Li et al., 2022) for 24 h, respectively. Cells treated with DMEM only were used as blank controls, and Dexra was used as a positive control. Finally, we also injected Mdivi-1 (5μM, in DMSO), an inhibitor of mitochondrial division (Nhu et al., 2021).

### Assessment of oxidative stress

Intracellular ROS levels were measured using the fluorescence dye DCFH-DA according to the manufacturer’s instructions. Briefly, DCFH-DA solution (1 μM) was prepared in PBS and added to H9c2 cells cultured in a 6-well plate before incubation at 37°C for 30 min. After incubation, the cells were washed with PBS. Fluorescent photomicrographs were taken at 20× magnification using a fluorescence microscope from Leica Microsystems, Ltd. ROS levels were quantified with a Beckman flow cytometer (Beckman, California, United States).

### Determination of enzymatic indices

The activity of SOD and NADH was measured using an activity assay kit according to the manufacturers’ instructions. The absorbance and luminescence were measured using a microplate reader (Perkin Elmer, Massachusetts, United States).

### Electron microscopy

Cells were fixed with 0.5% glutaraldehyde fixative at 4 °C for 15–30 min and were collected by centrifugation at 10,000–13,000 rpm for 5 min. The cells were further fixed with 3% glutaraldehyde at 4°C overnight and then treated with 1% osmium tetroxide at room temperature for 2 h. Thereafter, the samples were dehydrated in an acetone gradient, embedded in Epon 812, subjected to optical positioning, and cut into ultrathin sections. The sections were double-stained with uranyl acetate and lead citrate. The mitochondrial ultrastructure was examined using an H-7650 transmission electron microscope (Hitachi, Toyko, Japan).

### Determination of mitochondrial respiration

Approximately 10^6^ cells were used to measure mitochondrial respiration with an O2K respirometer (Oroboros Instruments, Austria). The oxygen concentration was determined and analyzed by using Oroboros DatLab 7.4 software. In brief, a leak respiratory state was recorded in cells alone, electron transfer was coupled to phosphorylation by the addition of 5 mM ADP, and state 3 respiration supported by complex I was recorded. Maximal state 3 respiration with parallel electron input from complex I and complex II was recorded through the addition of 10 mM succinate, and complex II-supported respiration was measured in the presence of 6.25 µM rotenone. Maximal electron transfer capacity was recorded in the presence of 5 µM carbonyl cyanide p-(trifluoro-methoxy) phenyl-hydrazone (FCCP). Finally, antimycin (Ama), which completes complex Ⅲ and blocks all electron transport, was administered, and the oxygen consumption rate was measured as nonmitochondrial oxygen consumption.

### Determination of changes in the mitochondrial membrane potential (ΔΨm)

5.5′,6.6′-Tetrachloro-1.1′,3.3′-tetraethylbenzimidazolylcarbocyanine iodide (JC-1) staining was used to assess the mitochondrial membrane potential in H9c2 cells. Briefly, according to the predetermined experimental conditions, H9c2 cells were washed with warm Dulbecco’s phosphate-buffered saline (DPBS) before being stained with 100 μL of 2 μM JC-1 solution (mixed in DMEM without phenol red) and incubated under standard cell culture conditions for 30 min in the dark. After incubation, the cells were washed with warm DPBS, and fluorescence photomicrographs were taken at 20× magnification using a Leica Microsystems CMS GmbH inverted fluorescence microscope (Leica Camera AG, Barnack, German).

### Immunofluorescence

To assess mitochondrial localization, cells grown on coverslips were washed with cold PBS and fixed with 4% paraformaldehyde for 15 min. Then, the cells were permeabilized with 0.5% Triton X-100 for 20 min and blocked with 5% goat serum for 30 min. The cells were incubated with primary antibodies overnight at 4°C and with secondary antibodies for 1 h. After washing three times, the cells were stained with 4′-6-diamidino-2-phenylindole (DAPI) and imaged with a confocal laser scanning microscope.

### ATP measurement

Additionally, the intracellular ATP concentration was measured by using an ATP assay kit following the manufacturer’s protocol. The cells were lysed in the lysis buffer by repeated pipetting and centrifuged at 4 °C and 13,000 g for 10 min. The supernatants were used for the analysis of ATP levels, and the protein concentrations were determined by the BCA assay. A volume of fifty microliters of supernatant was added to 100 μl of ATP detection solution, incubated at room temperature for 5min, and mixed immediately, and the luminescence was determined using a luminometer (Flex Station 3). The concentration of ATP was calculated according to a standard curve and converted into nmol/μg protein.

### Western blotting

Protein expression was evaluated by Western blotting and quantified with densitometry. The Dox and Dox + Brev-treated H9c2 cells in 10 cm cell culture dishes were lysed with the RIPA lysis buffer. Taking 5 mg of tissue and adding 10 ml of lysis buffer. The Bradford method was used to measure the protein concentration, utilizing BSA as a standard. Equivalent amounts of proteins were mixed with loading buffer (5X) and boiled at 95°C for 5 min. The proteins were then resolved by electrophoresis on 10%–12% SDS-polyacrylamide gels (SDS–PAGE) and transferred onto PVDF membranes. After blocking with 5% milk in TBST for 2 h at room temperature, the membranes were incubated overnight at 4°C with specific antibodies, such as rabbit polyclonal GAPDH. WB analysis of protein expression and phosphorylation was performed using antibodies against AMPK, Parkin, PINK1, Akt, PI3K, Tom20, and GAPDH was used as an internal control. Specific signals were visualized using the Bio-Rad gel doc system (Bio-Rad Laboratories, California, United States). The data are presented as the mean ± SD (*n* = 3) and quantified by ImageJ analysis.

### Animal husbandry

Eight-week-old female C57BL/6 mice (SiPeiFu, Beijing, China) were used in the experiments. The animals were housed in cages and on a 12-h light/12-h dark cycle at 50% humidity and 25°C ± 2°C. The animals were given free access to a standard pellet diet and water. Plasma was obtained by centrifugation at 200 g for 10 min at 4°C and kept at −80°C prior to extraction.

The investigation conforms to the *Guide for the Care and Use of Laboratory Animals* published by the US National Institutes of Health (NIH Publication No. 85–23, revised 1985).

### Animal experimental protocols

The mice were randomly assigned to six treatment groups, including a control group, model group (Dox group), three dose Dox + Brev groups, and Dox + Dexra group.

To mimic human therapeutic regimens, a cumulative dose of 12 mg/kg of Dox was administered *via* three weekly i.p. injections (4 mg/kg on days 0, 7, and 14) except for the control group.

To investigate the effect of Brev *in vivo,* mice were treated with Brev, followed by daily i.p. injection of 4.8, and 16 mg/kg of Brev for 3 weeks, and mice in the Dox + Dexra treatment group received 12 mg/kg Dexra by intraperitoneal injection for 3 weeks in addition to Dox treatment. (Alexandre et al., 2020). Brev and Dexra were treated after Dox injections. Survival was monitored daily and heart function was assessed by echocardiography, 3 weeks after the first injection of Dox (Li et al., 2018).

The control group was injected with the same volume of normal saline. The mice were sacrificed 1 week after Dox administration.

### Tumor studies

Mice were subcutaneously injected with 1×10^5^ 4T1 breast tumor cells. One week after cell injection, when the mean diameter of the tumors was greater than 2 mm, the mice were treated with Dox or breviscapine as described previously. Tumor size was measured twice a week for up to 4 weeks, and tumor volumes were calculated with the following equation: V = 4π/3×(L/2)2× (W/2), where V, L, and W represent the volume, length, and width of the tumor, respectively.

### ^18^F-fluorodeoxyglucose PET/CT imaging

Dox and breviscapine group mice (fasted overnight) were anesthetized with 2% isoflurane and injected with ∼11MBq/0.2 ml of fluorodeoxyglucose (FDG) PET *via* a tail vein, then returned to their cages. Forty minutes later, mice were anesthetized again with 2% isoflurane and placed on a stereotactic bed in a microPET Focus 220 (Siemens Company, Berlin, Germany). A 20 min static PET scan was then initiated. Afterwards, mice were imaged in a microCAT II (Siemens Company, Berlin, Germany) at an X-ray beam intensity of 180mAs and an X-ray tube voltage of 80 kVp for anatomical co-registration with the PET images.

### Patients

Following more than 4 h of fasting and ensuring that the blood glucose level was less than 120 mg/dl, all patients received an intravenous administration of ^18^F-FDG (5.5 MBq/kg). PET/CT scans were started 60 min after injection using a combined PET/CT biograph (Siemens Company, Berlin, Germany). All scans were performed in a three-dimensional model. A low-dose CT scan was obtained first for attenuation correction and anatomical correlation. The ethical clearance conforms to the Guide for the Approval Document of Ethics Committee published by the Affiliated Hospital of Qingdao University (QDU-HEC-2022057).

### Analysis of cardiac function and histopathology

Mouse heart tissue from each group was stored in 4% paraformaldehyde, embedded in paraffin wax, and cut serially into 4 mm sections. Tissue sections were deparaffinized *via* immersion in xylene (3 times, for 5 min each) and rehydrated using a descending series of alcohols (100%, 90%, 85%, and 75% alcohol, 5 min each). The sections were stained with hematoxylin and eosin for histological analysis. Biopsy samples were stained using Masson’s trichrome stain to investigate any morphological and fibrotic changes in the heart. Blue staining represented collagen accumulation. Immunohistochemistry was performed using the Histone Simple stain kits (Nichirei, Tokyo, Japan) according to the manufacturer’s instructions. The sections were treated for 15 min with 3% H2O2 in methanol to inactivate endogenous peroxidases and were then incubated at room temperature for 1 h with the primary antibodies IL-1, 1:100. The ultrastructure was then observed at 20× magnification using a Leica Microsystems’ CMS GmbH inverted fluorescence microscope (Leica Camera AG, Barnack, Germany).

### Measurement of glucose levels

Mice were maintained on a normal chow diet. On the day of experimentation, the mice were fasted for 6 h (beginning at 8 a.m.), and blood was collected *via* the tail vein for the measurement of glucose levels.

H9c2 rat cardiomyoblast cells were washed with phosphate-buffered saline (PBS) and centrifuged and ultrasonically crushed, and incubated under standard cell culture conditions for 10 min. After incubation, the absorbance was measured using a microplate reader (Perkin Elmer, Massachusetts, United States).

### Enzyme-linked immunosorbent assay

Mouse tissues were lysed with the lysis buffer. The samples were sonicated with a Qsonica homogenizer using 30 Hz pulses for 20s and then centrifuged at 12,000 g for 10 min. The supernatant was collected, aliquoted into 200 µl vials, and stored at −80°C. The protein concentrations of the samples were quantified by the BCA assay. The samples were analyzed using enzyme-linked immunosorbent assay kits for TNF-α, IL-1β, and IL-6 according to the manufacturer’s protocol. The optical density was measured at 450 nm by using a VICTOR Nivo™ microplate reader (Perkin Elmer, Massachusetts, United States).

### Determination of changes in the mouse metabolome

Blood samples were resuspended, incubated on ice, centrifuged, diluted to the final concentration, and centrifuged. Finally, the supernatant was injected into the LC–MS/MS system for analysis. UHPLC–MS/MS analyses were performed using a Vanquish UHPLC system (Thermo Fisher, Massachusetts, United States) coupled with an Orbitrap Q Exactive^TM^ HF mass spectrometer (Thermo Fisher, Massachusetts, United States). The identified metabolites were annotated using the KEGG database, HMDB database, and LIPID Maps database. Principal component analysis (PCA) and partial least square discriminant analysis (PLS-DA) were performed using metaX. We applied univariate analysis (*t*-test) to calculate the statistical significance (*p*-value). Metabolites with a VIP>1, *p* value < 0.05, and fold change ≥2 or ≤0.5 were considered to be differentially abundant metabolites.

### Statistical analysis

Statistical analyses were performed using ANOVA with IBM SPSS Statistics (V19.0, America). The data are presented as the mean ± SD (*n* = 6–9). *p* < 0.05 was considered significant.

## Results

### Anthracyclines reduce myocardial glucose metabolism and increase fatty acid utilization

**FIGURE 1:**
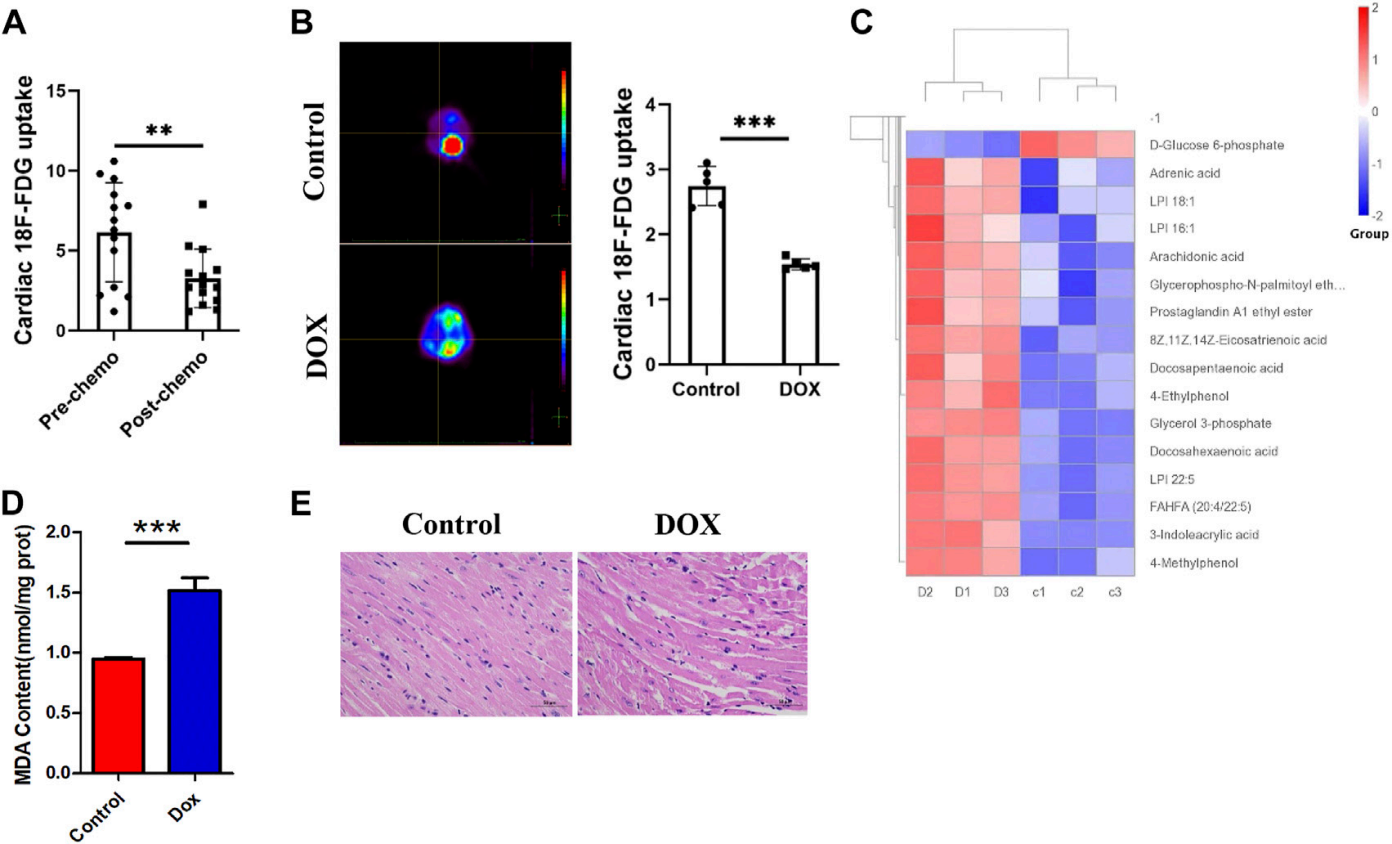
**(A)** Concentration of ^18^F-FDG taken up by the hearts of patients treated with chemotherapy and without chemotherapy. **(B)** ^18^F-FDG PET/CT image acquisition and cardiac ^18^F-FDG uptake. **(C)** Heat map of Dox-treated mice. **(D)** MDA content in H9c2 cells. **(E)** HE staining showing that Dox reduced the cardiomyocyte size. Control vs. Dox: ***p < 0.0005, **p < 0.005, *p < 0.05, mean ± SD. *n* = 6–9.

We first studied ^18^F-FDG PET/CT images of the hearts of patients treated with anthracycline chemotherapy and patients without chemotherapy, and data analysis showed (Figure 1A) a trend toward reduced myocardial glucose uptake in patients treated with chemotherapy. We predicted that changes in this parameter may be correlated with anthracycline-induced cardiotoxicity. To verify the intrinsic link between metabolic abnormalities and DIC, we established a Dox-induced DIC mouse model, and the ^18^F-FDG PET results showed that cardiac uptake was significantly lower in Dox-treated mice than in untreated mice after 4 weeks (Figure 1B). This suggests that anthracyclines affect myocardial glucose metabolism in both patients and mouse models.

Under normal conditions, the myocardium mainly uses sugars and lipids for energy supply, with sugars and lipids accounting for approximately 20%–30% and 60% of myocardial energy, respectively; furthermore, glucose is more efficient in producing ATP (Shao and Tian, 2015). How the heart adapts to the reshaping of energy metabolism in the myocardium by chemotherapeutic interventions and whether such alterations affect cardiac homeostasis and lead to the development of DIC are unclear. By analyzing metabolomic data from mice (Figure 1C), we found that the metabolites that showed increased levels after Dox exposure were mainly involved in lipid classification and that the decrease in the levels of glucose metabolism indicators such as D-glucose-6-phosphate and AKG was obvious. Moreover, KEGG enrichment analysis showed that lipid metabolic pathways were significantly altered after Dox exposure (Supplementary Figure S1). The level of the FAO metabolite MDA in the myocardium (Figure 1D) was significantly elevated after Dox treatment. This suggests that glucose metabolism in the heart is impaired after Dox intervention and is replaced by increased lipid burning, which is relatively less energy efficient. This inadequate lipid metabolism causes accumulation of a large amount of lipids in the myocardium, and pathological staining revealed a large number of lipid vacuoles in the hearts of Dox-exposed animals, leading to cardiac steatosis (Figure 1E).

### Anthracyclines increase myocardial oxygen consumption and decrease the ATP production rate

**FIGURE 2:**
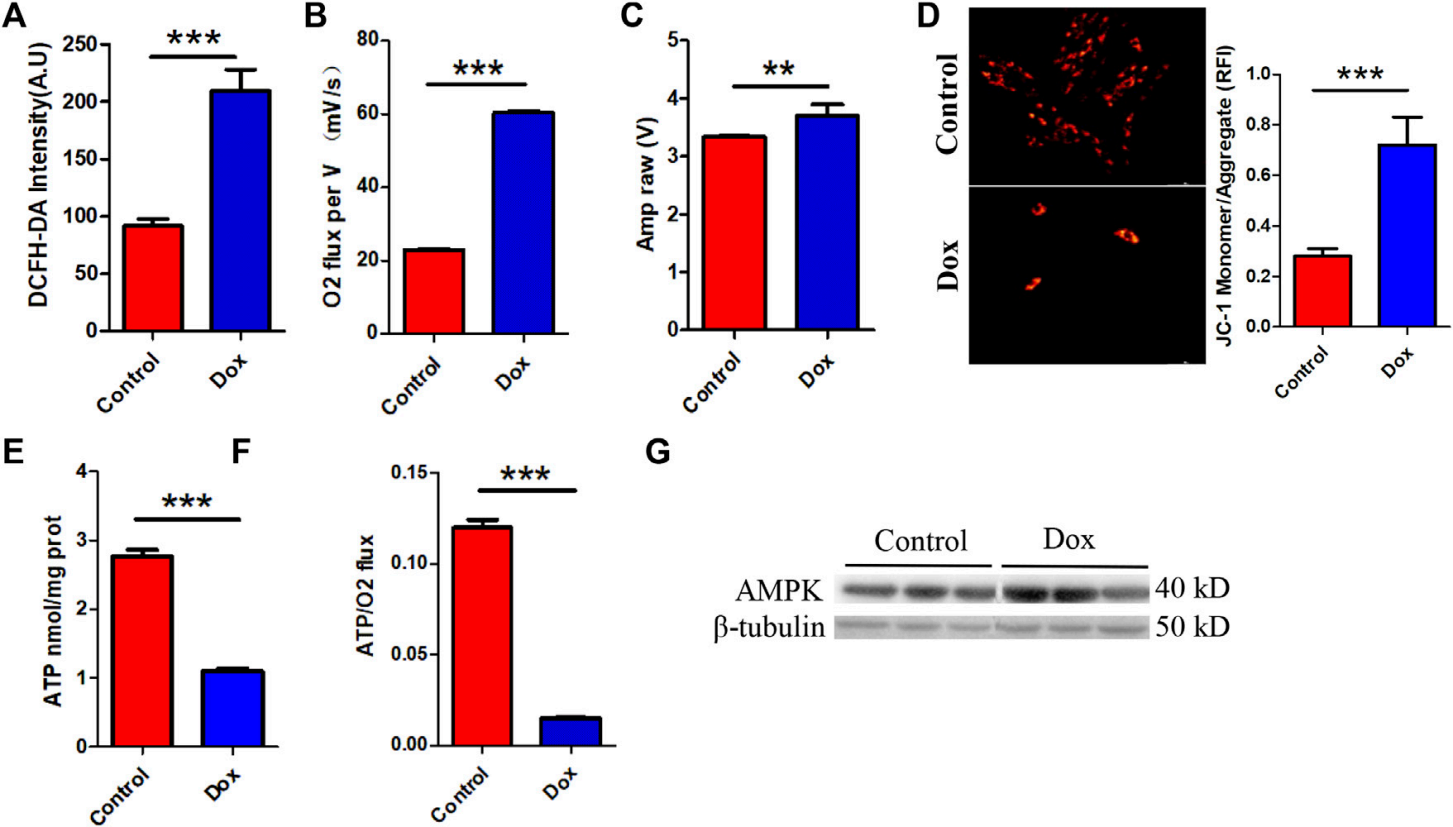
**(A)** Intensity of ROS staining in H9c2 cells. **(B)** Cardiac oxygen consumption in H9c2 cells. **(C)** The mitochondrial membrane potential. **(D)** JC-1 staining and bar graph. **(E)** ATP content in H9c2 cells. **(F)** The ATP production/oxygen consumption ratio in H9c2 cells. **(G)** The expression levels of AMPK and β-tubulin were measured *in vitro* by WB. Control vs. Dox: ***p < 0.0005, **p < 0.005, *p < 0.05, mean ± SD. *n* = 6–9.

Interestingly, this metabolic compensation increased myocardial ROS levels, leading to higher oxidative stress (Figure 2A). O2K energy metabolism analysis showed that Dox increased myocardial oxygen consumption (Figure 2B) and led to hyperpolarization of the mitochondrial membrane (Figure 2C) (this is also a sign of elevated levels of mitochondrial oxidative stress and is often seen in models of cardiac ischemia–reperfusion). JC-1 staining also showed that Dox exposure caused an increase in the membrane potential (enhanced red fluorescence intensity) in a few surviving cells (Figure 2D). Although compensatory processes such as increased oxygen consumption and lipid oxidation occurred in the heart, the ATP production rate in the myocardium was unfortunately drastically reduced in response to the reduction in glucose metabolism (Figure 2E). Comparison of the oxygen consumption rate and ATP production rate showed that Dox exposure led to the consumption of a large amount of oxygen by the myocardium but the production of only a small amount of ATP (Figure 2F), greatly increasing the burden on the heart. This deficit in energy output caused a significant increase in the level of the energy receptor AMPK in the Dox group (Figure 2G). Therefore, we hypothesize that the heart exhibits a high oxygen consumption rate and a low ATP production rate in response to impairment of glucose metabolism caused by anthracyclines and that this phenomenon does not fully meet the energy requirements of the myocardium but leads to excessive oxidative stress. It is therefore speculated that in response to the energy gap, lipid oxidation in the heart is increased and more ROS accumulates, and this poor feedback pathway may be an important driver of DIC.

### The new cardioprotective agent breviscapine promotes cardiac glucose metabolism and inhibits Dox-induced cardiomyopathy

**FIGURE 3:**
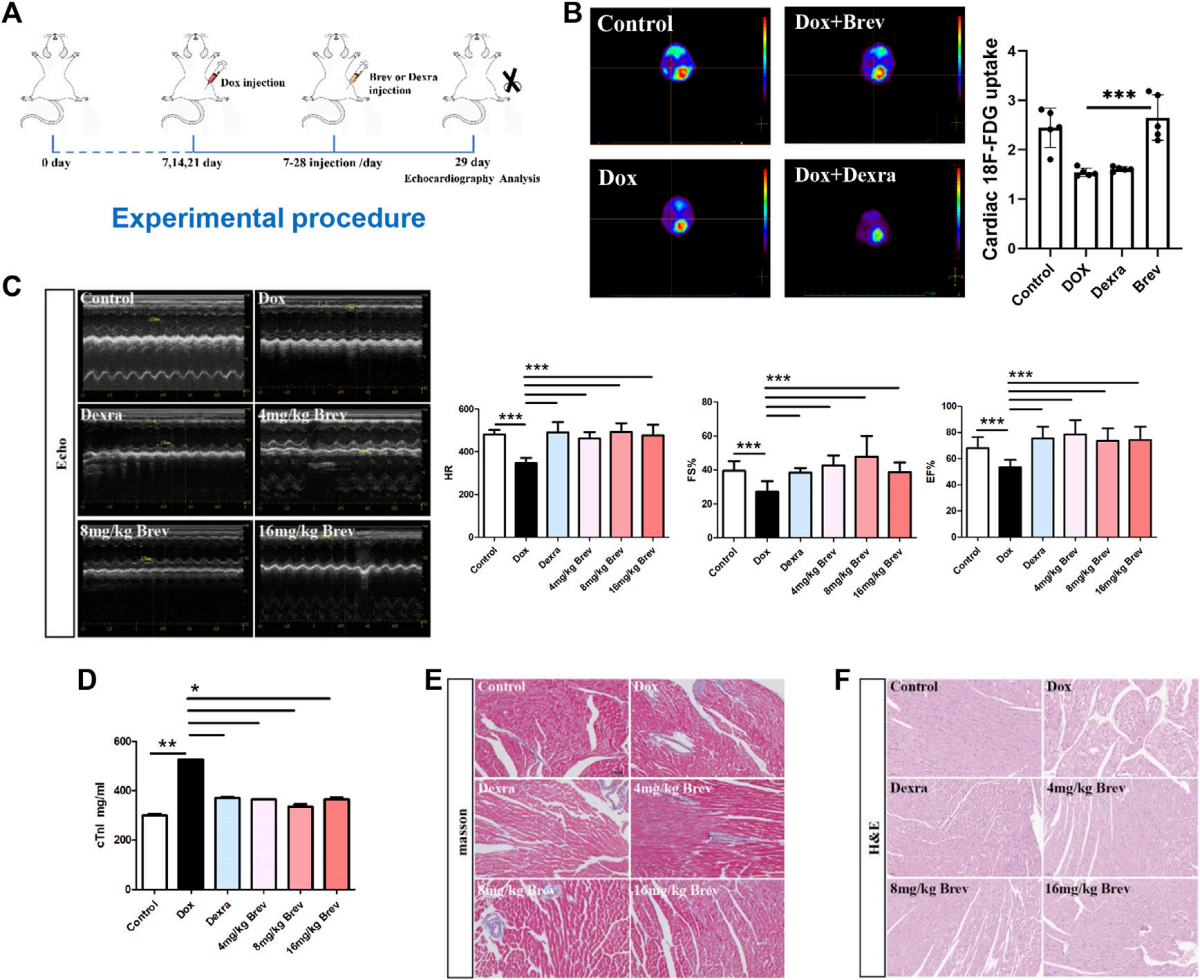
**(A)** Schematic protocol for mouse treatments. **(B)** ^18^F-FDG PET/CT imaging was performed using mice 1 week after the first Dox exposure, and the concentration of 18F-FDG taken up by the heart. **(C)** Echocardiograms showing that breviscapine prevented left ventricular dilatation induced by Dox. Representative M-mode short-axis echocardiograms showing that Dox induced left ventricular dilatation and that breviscapine exerted protected effects in the Dox + Brev group. The EF and FS in diastole were significantly lower in mice treated with breviscapine than in those treated with Dox, and the HR was significantly higher. **(D)** Breviscapine reduced the concentration of cTnI. **(E)** Masson staining showing that breviscapine reduced the degree of cardiac fibrosis induced by Dox. **(F)** HE staining showing that breviscapine protected against the reduction in cardiomyocyte size induced by Dox in the heart. Control vs. Dox, 4 mg/kg, 8 mg/kg, or 16 mg/kg Brev vs. Dox and Dexra vs. Dox: ***p < 0.0005, **p < 0.005, *p < 0.05, mean ± SD. *n* = 6–9.

Inspired by the aforementioned results, we investigated the protective effect of the flavonoid breviscapine, which is used to treat cardiovascular diseases, against the progression of DIC from a metabolic perspective. The experimental protocol is shown in Figure 3A, and ^18^F-FDG PET/CT imaging was performed using mice 1 week after the first Doxorubicin injection. As shown in Figure 3B, cardiac uptake was significantly higher in the breviscapine-treated group than in the Dox-treated group alone, suggesting that breviscapine intervention could reverse the Dox-induced decrease in cardiac glucose metabolism and remodel cardiac energy metabolism. Cardiac function and pathological changes after treatment with breviscapine were next examined, and the clinical drug dexrazoxane (Dexra) was used as a positive control. The echocardiography in Figure 3C showed that LVEF and LVFS in the Dox group were significantly lower than those in the control group, and LVESV and LVD were significantly higher than those in the control group. These results suggest that the left ventricular systolic function of the mice decreased after Dox treatment, and the Dox induced myocardial injury in this study was similar to dilated cardiomyopathy. Dox reduced systolic function, which is similar to what has been reported previously (Venturini et al., 1996; Sun et al., 2022). All functions were restored in the hearts of animals treated with Brev and Dox compared with those of animals treated with doxorubicin only. The cTnI is a biomarker of myocardial injury (Solaro and Solaro, 2020). The concentration of cTnI was significantly increased in mice treated with doxorubicin alone compared with those in the control group, and different concentrations of Brev showed better effects (Figure 3D). Moreover, the staining of pathological cardiac tissues after different treatments showed that the cardiac tissue structure in mice in the control group was normal. Unlike those in the control group, mice in the Dox group exhibited a large number of cardiac lesions, including necrosis, intracellular edema, myofibrillary disorder and rupture, and wavy degeneration of cardiac fibers. Interestingly, preconditioning with breviscapine prevented this type of injury, alleviated excessive accumulation of cardiac lipids, and restored myocardial homeostasis (Figure 3F). Masson staining of the myocardium showed that interstitial fibrosis was significantly increased after Dox treatment and that Brev intervention reduced the degree of cardiac fibrosis (Figure 3E). Cardiac ultrasound data showed that the LVESV and LVIDs were significantly higher in the Dox group than in the control group, suggesting ventricular cavity diameter enlargement and that breviscapine treatment could significantly alleviate these changes. These results suggest that breviscapine can reshape cardiac metabolism, increase cardiac glucose uptake, reduce cardiac steatosis, and alleviate doxorubicin-induced cardiac dysfunction and changes in myocardial morphology.

### Breviscapine remodels cardiac glucose and lipid metabolism by regulating peripheral serum levels and myocardial AKT expression

**FIGURE 4:**
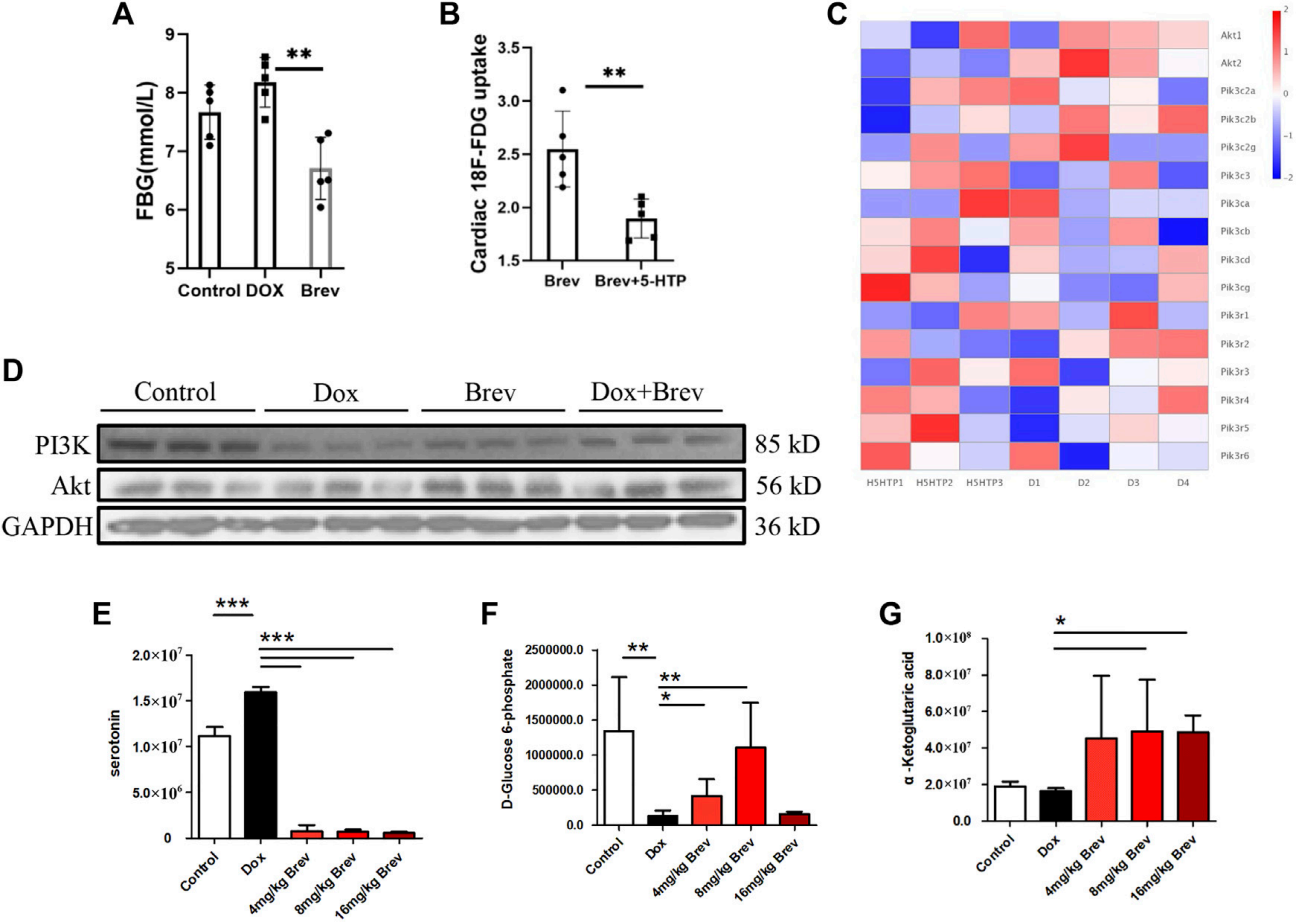
**(A)** Fasting blood glucose levels in mice. **(B)** Concentration of ^18^F-FDG taken up by the heart after supplementation with 5-HT. **(C)** The heat map of transcriptome analysis after supplementation with 5-HT. **(D)** The expression levels of PI3K, Akt, and GAPDH were measured *in vitro* by WB. **(E)** The level of serotonin in the peripheral serum in mice. **(F)** The level of D-glucose 6-phosphate in mice. **(G)** The level of α-ketoglutaric acid in mice. Control vs. Dox, 4 mg/kg, 8 mg/kg, or 16 mg/kg Brev vs. Dox and Brev+5-HT vs. Brev: ***p < 0.0005, **p < 0.005, *p < 0.05, mean ± SD. *n* = 6–9.

Next, we studied how breviscapine regulates the balance of sugar and fat metabolism. As shown in Figure 4E, the level of serotonin in the peripheral serum in breviscapine-treated mice was dramatically increased compared with that in Dox-treated mice, and the degree of the decrease showed a linear relationship with the dose of breviscapine, with a decrease of more than 95% being observed in the high-dose group. Serotonin (5-HT) is a monoamine that has a variety of functions in neuronal and nonneuronal systems (Okaty et al., 2019). In the central nervous system, 5-HT acts as a neurotransmitter that regulates mood and feeding behavior (Berger et al., 2009). Recent studies have shown that peripheral 5-HT plays an important role in the metabolic regulation of peripheral tissues by inhibiting adaptive thermogenesis in brown adipose tissue. Inhibition of 5-HT synthesis reduces weight gain and improves metabolic dysfunction in a diet-induced obesity mouse model (Zemdegs et al., 2016; Xia et al., 2021). Genome-wide association studies have also revealed a genetic link between the serotonergic system and obesity. Serotonin has two different effects on glucose metabolism: it directly induces insulin secretion in pancreatic B cells, lowering blood glucose levels; and it inhibits the uptake of glucose from the blood by tissues other than the liver and skeletal muscle, raising blood sugar levels (Watanabe et al., 2011; Carmean et al., 2019; Yabut et al., 2019). Studies have shown that serotonin treatment can lead to a sudden and large increase in blood glucose levels, but how serotonin inhibits tissue uptake of glucose from the blood remains unclear. Our experimental results showed that after breviscapine treatment, peripheral serotonin levels decreased sharply, corresponding to an increase in cardiac glucose intake (Figure 4E), while liver glucose intake did not change significantly (Supplementary Figure S2) and fasting glucose (FBG) levels were decreased (Figure 4A). In the breviscapine group, additional supplementation with 5-HT increased the level of serotonin in mice and decreased cardiac glucose uptake (Figure 4B). To further analyze how serotonin regulates cardiomyocyte metabolism, we added additional 5-HT to increase serotonin levels in Dox-induced myocardial injury model mice and then collected the hearts of the mice for transcriptome analysis. The results showed that oxidation of fatty acids in the group treated with Dox and 5-HT was significantly higher than that in the Dox group alone (Supplementary Figure S3). We then analyzed the changes in the PI3K-protein kinase B (Akt/PKB) pathway, an important regulator of myocardial glucose metabolism that plays an important role in regulating glucose uptake; promoting cell division, proliferation, and survival; and inhibiting apoptosis. The results showed that the addition of 5-HT directly inhibited the gene expression of Akt but did not significantly change the PI3K catalytic subunit or regulatory subunit level (Figure 4C). Loss of AKT reduces the ability of cardiomyocytes to metabolize glucose. These results suggest that breviscapine relieves the inhibitory effect of serotonin on the tissue glucose uptake by significantly reducing the level of serotonin and ensures the absorption of glucose from the blood under pathological conditions, revealing the possibility that the body may participate in the regulation of cardiac metabolism remodeling through neurotransmitters. More importantly, breviscapine, as a commonly used cardiovascular drug in China, can reduce peripheral serotonin levels by more than 90% and has a more robust effect than the classic serotonin reuptake inhibitor fluoxetine in the treatment of myocardial hypertrophy and heart failure induced by myocardial glucose metabolism disorder and diabetes, representing a new safe agent for the treatment of Dox-induced cardiotoxicity. Epidemiological studies have also shown that diabetes increases the risk of Dox-induced cardiotoxicity; thus, this study provides a potential treatment strategy for patients with diabetes and cancer (Chang et al., 2021).

**FIGURE 5:**
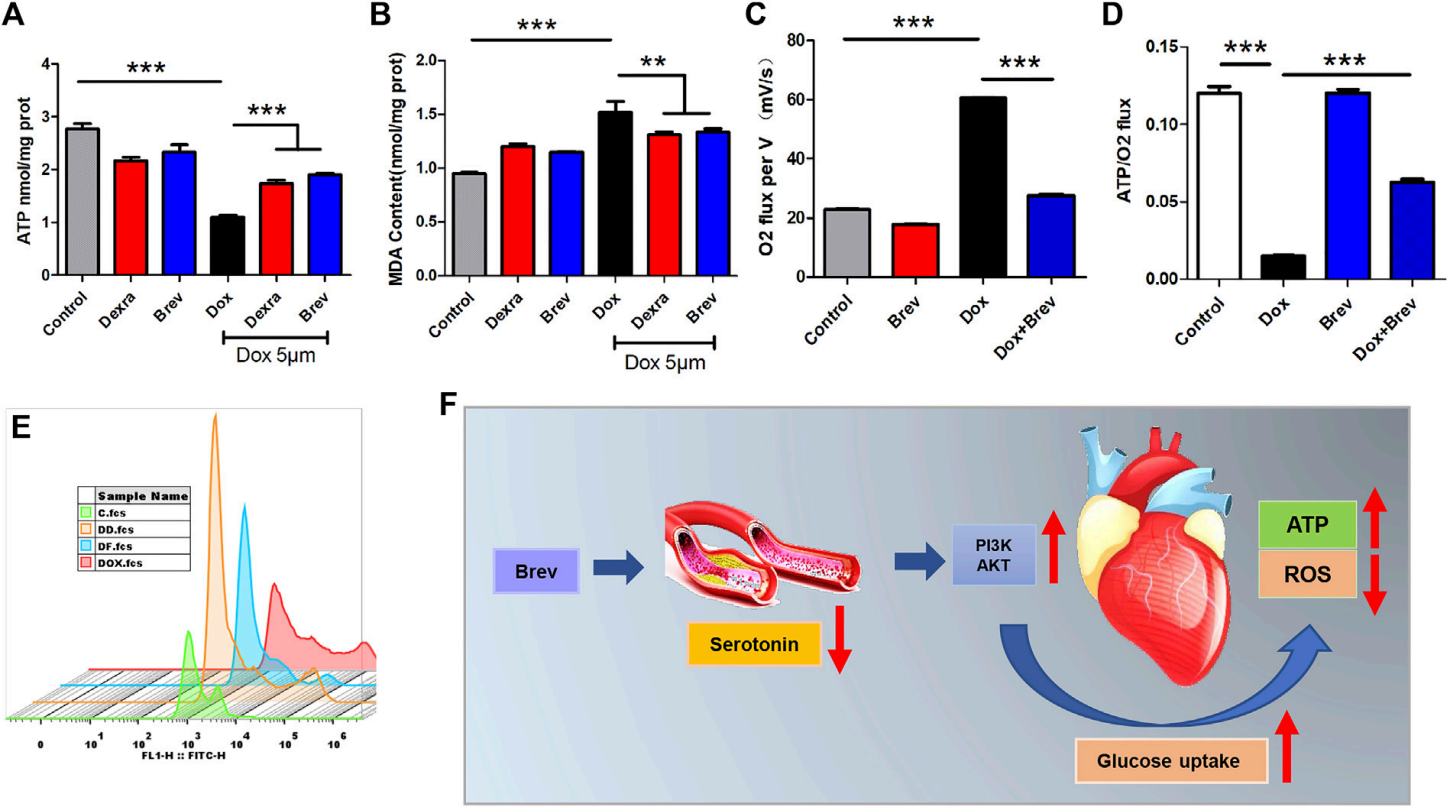
**(A)** ATP content in H9c2 cells. **(B)** MDA content in H9c2 cells. **(C)** Cardiac oxygen consumption in H9c2 cells. **(D)** The ATP production/oxygen consumption ratio in H9c2 cells. **(E)** The intensity of ROS staining in H9c2 cells. **(F)** Schematic diagram. Control vs. Dox, Brev vs. Dox, and Dexra vs. Dox: ***p < 0.0005, **p < 0.005, *p < 0.05, mean ± SD. *n* = 6–9.

Based on these findings, we investigated how breviscapine regulates glucose. Utilization by the heart. The results showed that PI3K protein expression in Dox-exposed mice was significantly decreased and that the PI3K protein expression level in cardiac tissue was significantly increased after breviscapine intervention. Interestingly, AKT expression was maintained at low levels in both the control and Dox-exposed groups, and significantly increased AKT expression was observed after breviscapine treatment (Figure 4D). Previous studies have shown that AKT stimulates glucose metabolism in cells, converting glucose to D-glucose 6-phosphate *via* hexokinase for glycolysis or polymerization to glycogen. Our results showed that after breviscapine promoted the expression of myocardial AKT, glucose metabolism-related D-glucose 6-phosphate and α-ketoglutaric acid levels were significantly increased (Figure 4F,G), suggesting that breviscapine enhanced the ability of the myocardium to use glucose as an energy source through AKT and promoted glucose catabolism. Previous studies have reported that increased PI3K-Akt expression can promote sugar utilization in peripheral tissues, reduce insulin resistance, enhance cardiac energy supply, and prevent cardiac insufficiency, while the inhibition of the PI3K-Akt pathway affects the physiological activities of cardiac cells (Zhu et al., 2013). These results suggest that breviscapine enhances resistance to Dox-induced myocardial injury by modulating the activation of the PI3K-Akt pathway. These results suggest that breviscapine regulates blood glucose levels by inhibiting serotonin, blocks the restriction of glucose utilization in the myocardium, and promotes glucose metabolism in the myocardium by activating the PI3K-Akt pathway. Therefore, serotonin may be an important target for regulating cardiac glycolipid metabolism. We then analyzed changes in cardiac function and homeostasis following remodeling of myocardial glycolipid metabolism. Compared with that in the Dox model group, myocardial ATP production in the breviscapine-treated group was significantly increased (Figure 5A), while the levels of ROS and MDA, a metabolite of FAO, were significantly decreased (Figures 5B,E). O2K energy metabolism analysis showed that breviscapine inhibited the dramatic increase in cardiac oxygen consumption caused by Dox exposure (Figure 5C), significantly decreased the ATP production/oxygen consumption ratio (Figure 5D), and normalized the mitochondrial membrane potential (Supplementary Figure S4). This suggests that breviscapine treatment increases the myocardium’s ability to use high-energy efficient glucose as an energy source; improves the efficiency of ATP production; and reduces cardiac load, oxidative stress, and myocardial oxygen consumption. In conclusion, breviscapine can effectively break the vicious cycle of a high oxygen consumption rate and inadequate lipid combustion in the heart caused by Dox exposure by regulating the serotonin-glycemia-myocardial AKT loop, remodeling myocardial energy metabolism, increasing glucose utilization, and reducing FAO (Figure 5F), preventing the further development of DIC.

### Breviscapine clears mitochondrial damage and restores myocardial homeostasis after Dox treatment through the PINK1/Parkin signaling pathway

Although hypotheses regarding the mechanisms of DIC have varied over the past decades, the key features of DIC are redox imbalance and impaired mitochondrial function. The results prevented previously improved the hypothesis of DIC development from the perspective of the cardiac energy ratio and energy output efficiency but raise a key question, namely, how breviscapine achieves efficient energy production and cardiac microenvironmental homeostasis by optimizing the metabolism network in the heart after mitochondrial injury. Thus, we aimed to answer this question. The Pten-induced putative kinase1 PINK1/Parkin signaling pathway is one of the major pathways mediated by mitochondrial autophagy and plays an important role in maintaining the energy metabolism of cardiomyocytes (Dewanjee et al., 2021). When mitochondria in myocardial cells are damaged, PINK1, as the “sentinel” of the mitochondrial quality control system, accumulates in the outer membrane of mitochondria and collects and phosphorylates Parkin (Poole and Macleod, 2021). Activated Parkin ubiquitinates damaged mitochondria, which then fuse with lysosomes to complete degradation. Enhanced mitochondrial autophagy mediated by the PINK1/Parkin signaling pathway can maintain mitochondrial homeostasis in cardiomyocytes and slow the progression of heart disease (Dorn, 2016).

**FIGURE 6:**
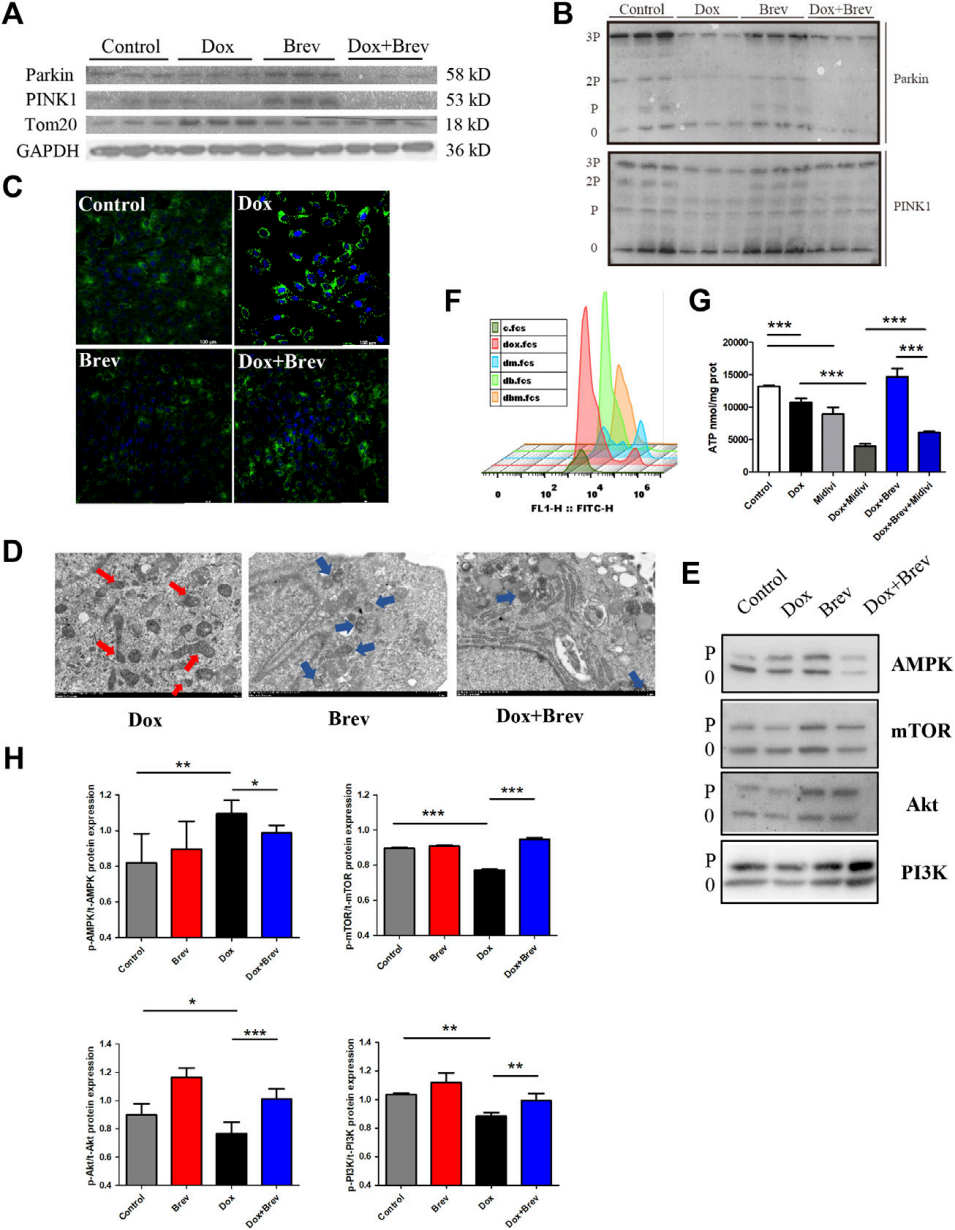
**(A)** Expression levels of PINK1, Parkin, Tom20, and GAPDH were measured *in vitro* by WB. **(B)** The expression levels of p-PINK1, p-Parkin, and Tom20 were assessed. **(C)** Mitotracer staining. **(D)** Electron microscopy. **(E)** The expression levels of p-AMPK, p-mTOR, p-Akt, and p-PI3K were evaluated. **(F)** The intensity of ROS staining in H9c2 cells after supplementation with Mdivi-1. **(G)** ATP content in H9c2 cells after supplementation with Mdivi-1.**(H)** Bar graph of p-AMPK/t-AMPK, p-mTOR/t-mTOR, p-Akt/t-Akt, and p-PI3K/t-PI3K. Control vs. Dox, Dox + Mdivi-1 vs. Dox, and Dox + Brev + Mdivi-1 vs. Dox + Brev: ***p < 0.0005, **p < 0.005, *p < 0.05, mean ± SD. *n* = 6–9.

Therefore, we speculated that breviscapine enhances mitochondrial autophagy through the PINK1/Parkin pathway and maintains mitochondrial homeostasis in cardiomyocytes. WB showed that compared with that in the Dox group, the total protein expression level of PINK1/Parkin in the breviscapine treatment group was significantly increased (Figure 6A), and the corresponding phosphorylation level was also significantly increased (Figure 6B). In the Dox treatment group, PINK1/Parkin protein expression showed a decreasing trend, and protein phosphorylation was significantly decreased, while the level of the mitochondrial membrane protein Tom20 was increased, suggesting that although Dox induced mitochondrial damage, it inhibited the timely clearance of damaged mitochondria. A large number of broken and damaged mitochondria were observed in the Dox group (red arrow, Figure 6D), while a large amount of lysosomal-wrapped mitochondrial autophagy was observed in the Brev group (blue arrow). When Dox and Brev were applied simultaneously, the mitochondrial ridge was obvious, mitochondrial morphology was normal, and the membrane structure was intact. Upon staining with MitoTracker, the highest fluorescence intensity was found in the Dox group (Figure 6C), indicating that there were significantly more mitochondria in the Dox group than in the control and Brev groups. Moreover, the levels of the antioxidative proteins NADH and SOD were significantly increased after Brev treatment (Supplementary Figure S5). Phosphorylation of the energy receptor AMPK was significantly reduced in the Brev treatment group compared with the Dox group, while cell growth control centers were activated (Figure 6E). These results indicate that Dox exposure causes myocardial damage, mitochondrial accumulation, and homeostasis imbalance, which to some extent explains why cardiac energy output efficiency is reduced and oxidative stress is increased after Dox administration. Breviscapine can promote mitochondrial autophagy, remove damaged mitochondria, and restore myocardial homeostasis, effectively ensuring the dynamic balance between cardiac ATP production and oxidative stress.

When this effect of breviscapine was blocked by Mdivi-1, the ROS level in cardiomyocytes was significantly increased (Figure 6F), while ATP production was significantly decreased (Figure 6G). Furthermore, the survival rate of cardiomyocytes was decreased (Supplementary Figure S6), and the reparative effect of breviscapine on cardiomyocytes was inhibited. When Dox and Mdivi-1 were combined, ATP production and the survival rate of myocardial cells were further reduced, the ROS level was increased, and myocardial injury was aggravated. These results suggest that mitochondrial autophagy plays a key role in Dox-induced cardiomyocytes injury. Metabolic network remodeling of the heart alone cannot completely offset the dysfunction caused by mitochondrial injury, and the myocardial protective effect of breviscapine depends on the regulation of glucose and lipid metabolism and the activation of mitochondrial autophagy. This may also be the reason why regulation of the cardiac metabolic network has not been considered in regards to the hypothesis of DIC development. It is suggested that preventing the progression of the disease and restoring cardiac function should be equally important in the treatment of the symptoms of cardiac dysfunction and heart failure caused by Dox, which is more beneficial for prognosis and the quality of life of patients.

### Breviscapine synergizes with breast cancer chemotherapy by alleviating inflammation and reducing immune-suppressive cell infiltration after Dox administration

**FIGURE 7:**
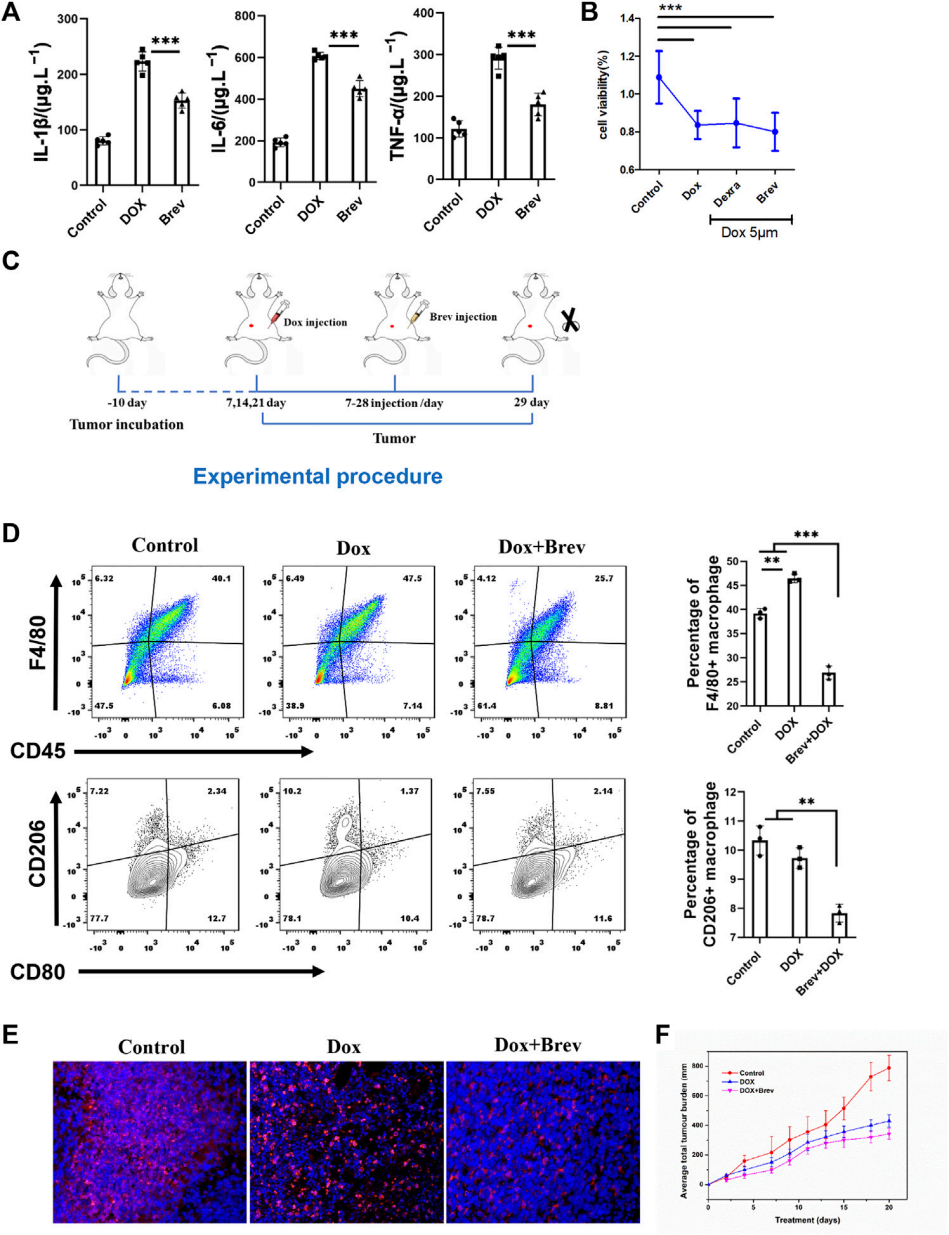
**(A)** Serum levels of the inflammatory cytokines IL-1β, IL-6, and TNF-α. **(B)** MCF-7 cell viability *in vitro*. **(C)** Schematic protocol of the treatment of mice with tumors. **(D)** Mouse tumors were collected for FCM. **(E)** Histological staining of tumors for the M2 phenotypic marker CD206. **(F)** The average of total tumor burden. *p < 0.0005. Control vs. Dox, Dexra vs. Dox, and Brev vs. Dox: ***p < 0.0005, **p < 0.005, *p < 0.05, mean ± SD. *n* = 6–9.

According to the theory of mitochondrial origin, mitochondria are derived from the fusion of aerobic bacteria, meaning that mitochondria have foreign properties (Gray, 2012). When mitochondria are damaged, damage-associated molecular patterns (DAMPs) are triggered, activating the body’s immune response (Wilkins et al., 2017). Therefore, we hypothesized that breviscapine promotes the clearance of damaged mitochondria and helps reduce inflammation. The results showed that the serum levels of the inflammatory cytokines interleukin-1β (IL-1β), IL-6, and tumor necrosis factor α (TNF-α) were significantly decreased after breviscapine intervention (Figure 7A), and immunostaining of heart tissues showed that IL-1β was expressed at low levels (Supplementary Figure S7). These results suggest that breviscapine can reduce the secretion of inflammatory factors and reduce the inflammatory response in Dox-exposed mice. Inflammatory regulatory factors and effector cells are important components of the tumor microenvironment and play an important role in the relationship between inflammation and tumors (Elinav et al., 2013; Sautes-Fridman et al., 2019). Inflammation in the tumor microenvironment has a variety of protumor effects, which can promote the proliferation and survival of malignant cells, angiogenesis, and metastasis; weaken the body’s acquired immune response; and change the body’s response to hormones and chemotherapy drugs (Iyengar et al., 2016; Munn and Mellor, 2016). Breviscapine reduced inflammation after Dox treatment but did not affect the killing effect of Dox on breast cancer cells *in vitro* (Figure 7B). Whether it can help prevent the migration of inflammatory cells to the tumor focus, especially in the cooperative treatment of tumor-associated macrophages (TAMs), remains unclear. TAMs are important infiltrating immune cells and can account for 50% of tumor cells in breast cancer. TAMs and related cells in mouse and human tumors are usually M2 macrophages, which have promoted tumor growth and angiogenesis, enhanced treatment resistance, and inhibited acquired immunity (Chen et al., 2019; Pan et al., 2020). Reducing TAM infiltration or drug-induced clearance of TAMs is an emerging strategy for tumor immunotherapy. Thus, we constructed a 4T1 breast cancer mouse model (Figure 7C). Mice with 4T1 tumors *in situ* (200 mm^3^) were randomly divided into 3 groups (*n* = 5) and injected with phosphate-buffered saline (PBS), Dox, or Dox + Brev. Dox and Brev were administered at doses of 10 mgkg^−1^ body weight and 1.4 mgkg^−1^ body weight, respectively. The mice were sacrificed 7 days after treatment, and the tumors were collected for FCM and histological evaluation. After Dox + Brev treatment, the proportion of tumor-associated macrophages (TAMs) infiltrating the tumor was reduced (Figure 7D). Histological staining of tumor tissue showed significantly lower expression of the M2 phenotypic marker CD206 (Figure 7E). The tumor volume in mice treated with Dox + Brev was significantly lower than that in mice treated with Dox alone (Figure 7F), suggesting that breviscapine can inhibit tumor growth by reducing inflammation and TAM infiltration.

## Discussion

This study reveals that glucose and lipid metabolism dysfunction are an important driving force of anthracycline-induced cardiotoxicity and that restoring cardiac metabolism, promoting myocardial glucose metabolism, and improving myocardial energy output efficiency are effective methods for preventing the cardiac side effects of Dox. They can promote mitochondrial autophagy, the removal of damaged mitochondria. Reducing inflammation enhances the anticancer effect of Dox.

The mammalian heart uses a large amount of energy for continuous contraction, and the tight coupling between ATP production and cardiac contraction is essential for proper heart function (Bertero and Maack, 2018; Timm et al., 2018). This process requires precise regulation by the cardiac energy metabolism network. The most striking feature of this network is its metabolic flexibility in response to a variety of stimuli, including developmental changes and nutritional status, and the ability of the heart to reshape metabolic pathways to regulate myocardial energy and systolic function under chronic pathophysiological conditions (Bertero and Maack, 2018; Kim et al., 2018). This metabolic remodeling contributes to the preservation of cardiac function or accelerates the progression of disease. There are many studies on cardiac diseases, such as cardiac hypertrophy and heart failure, but there has been little research on DIC. Previous research studies have focused on ROS production and topoisomerase II-β targeting, which result in DNA damage, mitochondrial dysfunction and, Ca^2+^ mishandling. However, ROS-clearing iron chelators and the topoisomerase II-β modulator dexra fail to provide significant benefits, suggesting that other factors are also involved. How Dox regulates the metabolic network in the myocardium, how remodeling of this metabolic network affects the energy output efficiency of the heart, and whether Dox promotes cardiotoxicity remain unknown. Recent studies have revealed that ferroptosis of cardiomyocytes caused by lipid peroxidation accumulation plays an important role in the progression of DIC, suggesting that Dox-exposed cardiomyocytes have a tendency to enhance lipid metabolism (Xie et al., 2021; Kitakata et al., 2022). Our findings further elucidate that Dox-induced cardiotoxicity is driven by a vicious feedback loop of dysregulation of glucose and lipid metabolism and a resulting increase in oxygen consumption, inefficient energy production, and increased levels of oxidative stress.

Breviscapine is the most widely used classical drug for the treatment of cardiovascular and cerebrovascular diseases in China, and it is included in the Chinese Pharmacopoeia and National Essential Drugs List and is an essential medicine for emergency treatment in Chinese hospitals. Our study found that breviscapine improves the efficiency of ATP production by regulating the serotonin-glycemic-myocardial AKT circuit, reshaping myocardial energy metabolism, increasing glucose utilization, and reducing lipid oxidation. It alleviates the effects of low ATP production, high oxygen consumption, and high oxidative stress caused by inadequate metabolism of large amounts of lipids in the heart caused by Dox exposure, reduces the expression of AMPK, blocks the vicious cycle of excessive lipid uptake in the myocardium, and slows the development of DIC (Kolwicz and Tian, 2011). In addition, our results suggest that serotonin may act as a supplementary branch of insulin to regulate blood glucose, which supplies blood glucose to the heart and other important organs under pathological conditions. Serotonin has contradictory effects on blood glucose regulation. On the one hand, it interacts with insulin to lower blood glucose levels; on the other hand, it causes tissues other than the liver and skeletal muscles to absorb glucose and raise blood sugar levels. This suggests that lowering peripheral serum levels, such as through breviscapine treatment, may aid the use of glucose by organs. In addition, the inhibitory effect of breviscapine on cardiotoxicity depends on the enhancement of mitochondrial autophagy, and cardiac metabolic remodeling alone cannot lead to clearance of accumulated mitochondria in the context of cardiac injury caused by Dox. Breviscapine induces autophagy *in vivo* to restore myocardial mitochondrial homeostasis through the PINK1/Parkin pathway, alleviating the cardiotoxicity caused by doxorubicin. Considering that the activation of autophagy is involved in the occurrence and development of a variety of diseases, breviscapine may be a candidate drug for mitochondrial autophagy-related diseases, giving it very good clinical application value. Recent studies have shown that breviscapine has certain inhibitory effects on tumor growth and the biological behavior of tumor cells (Ye et al., 2020). The results of this study showed that breviscapine can prevent doxorubicin-induced cardiac dysfunction in breast cancer tumor models and reduce the levels of inflammatory factors and immunosuppressive macrophage infiltration by eliminating damaged mitochondria, exerting an effect that is synergistic with the antitumor effect of doxorubicin. Breviscapine reduces tumor growth by providing a dual therapeutic advantage in cancer, preventing the cardiotoxicity of anthracyclines. Breviscapine protects against anthracycline-induced cardiotoxicity and exerts a synergistic antitumor effect against anthracyclines, giving it good clinical application value.

## Data Availability

The original contributions presented in the study are included in the article/Supplementary Material; further inquiries can be directed to the corresponding author.
