# Supplementary material for: Breviscapine remodels myocardial glucose and lipid metabolism by regulating serotonin to alleviate doxorubicin-induced cardiotoxicity
Source: Front Pharmacol. 2022 Sep 27;13:930835. doi: 10.3389/fphar.2022.930835 (PMC9551275; doi:10.3389/fphar.2022.930835)

Supplementary Material

**Supplementary Figures Legends**

**Supplementary Figure 1.** Dox promotes lipid metabolic pathway significantly altering. The KEGG pathways of target metabolites of negative ESI mode of Dox. The functions of these metabolites and metabolic pathways were studied using the KEGG database. The metabolic pathways enrichment of differential metabolites was performed, when the ratio was satisfied by x/n > y/N, a metabolic pathway was considered as enrichment, when *P*-value of metabolic pathway < 0.05, metabolic pathway was considered as statistically significant enrichment. The Y-axis is *P*, representing the significance level of this pathway. The larger P, the redder the node color. The X-axis is Pathway impact, indicating the importance of the metabolic pathway. The larger the value is, the longer the node radius is.

**Supplementary Figure 2.** Breviscapine doesn’t change significantly liver glucose intake. A. ^18^F-FDG PET/CT imaging of the liver. B. GLU content uptake of H9c2 cells. Control vs DOX and Brev vs DOX: ***P*<0.005, mean ± SD. n=6-9.

**Supplementary Figure 3.** 5-HT significantly increases oxidation of fatty acids than DOX group alone. Transcriptome analysis in DOX-induced myocardial injury model mice added an additional 5-HT. For clustering heat maps, the data were normalized using z-scores of the intensity areas of differential gene and were plotted by P heatmap package in R language. The correlation between differential gene was analyzed by cor () in R language (method=pearson). Statistically significant correlations between differential genes were calculated by cor. mtest() in R language. *P*-value < 0.05 was considered as statistically significant and correlation plots were plotted by corrplot package in R language.

**Supplementary Figure 4.** Breviscapine promotes normalized mitochondrial membrane potential. (A) The effect of Brev on ΔΨm by JC-1 staining in H9c2 cells.

(B) Bar graph of JC-1 staining in H9c2 cells. (C) O2k evaluated the mitochondrial membrane potential. Control vs DOX and Brev vs DOX: ***P*<0.005, mean ± SD. n=6-9.

**Supplementary Figure 5.** Breviscapine significantly increases the levels of the antioxidative proteins NADH and SOD. Control vs DOX, Dexra vs DOX, and Brev vs DOX: *** *P*<0.0005, ***P*<0.005, * *P*<0.05, mean ± SD. n=6-9.

**Supplementary Figure 6.** Mdivi inhibits the reparative effect of breviscapine on cardiomyocytes. The cell viability after Mdivi inhibitors by CCK8 kits. Control vs DOX, DOX+Mdivi vs DOX, and DOX+Brev+Mdivi vs DOX+Brev: *** *P*<0.0005, ***P*<0.005, * *P*<0.05, mean ± SD. n=6-9.

**Supplementary Figure 7.** Dox promotes IL-1β expression of heart tissues and breviscapine inhibits the increase.

Suppl Figure1.


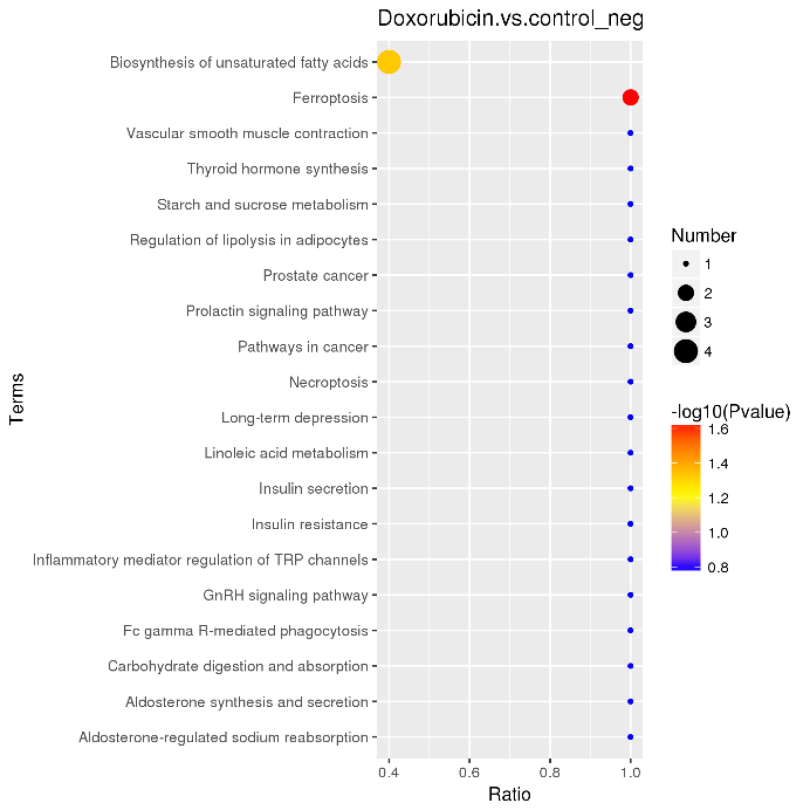


Suppl Figure2.


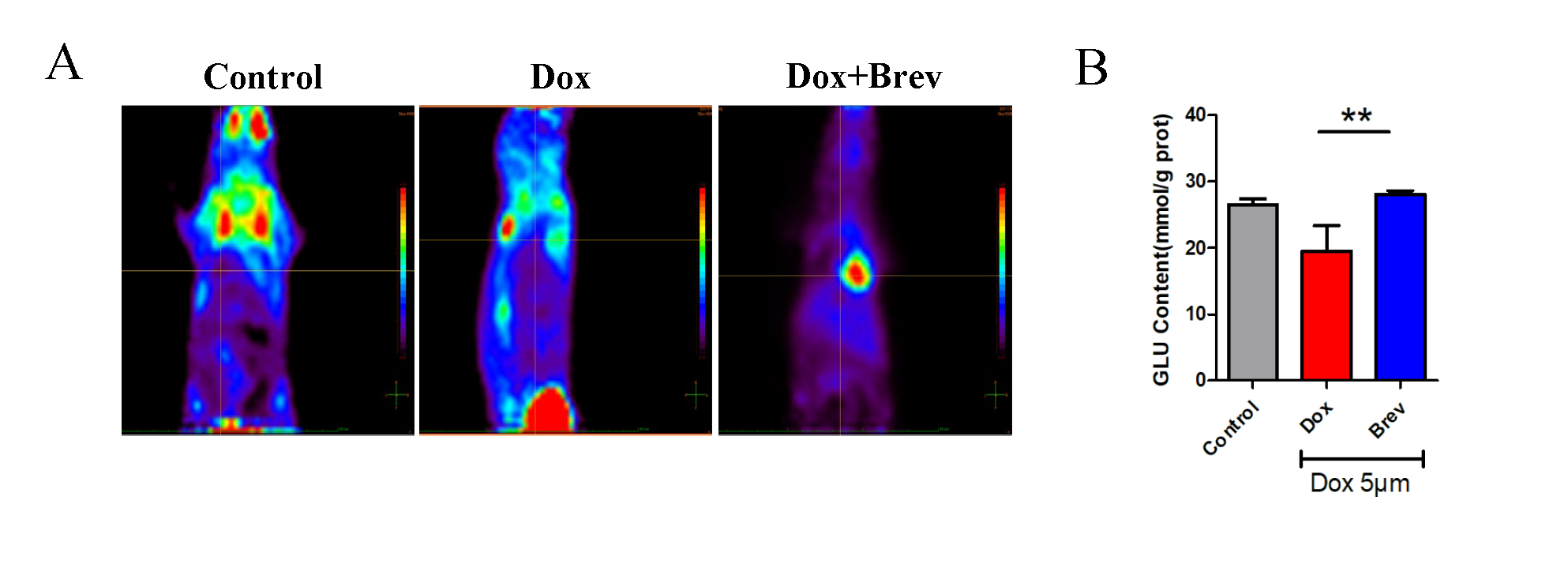


Suppl Figure3.


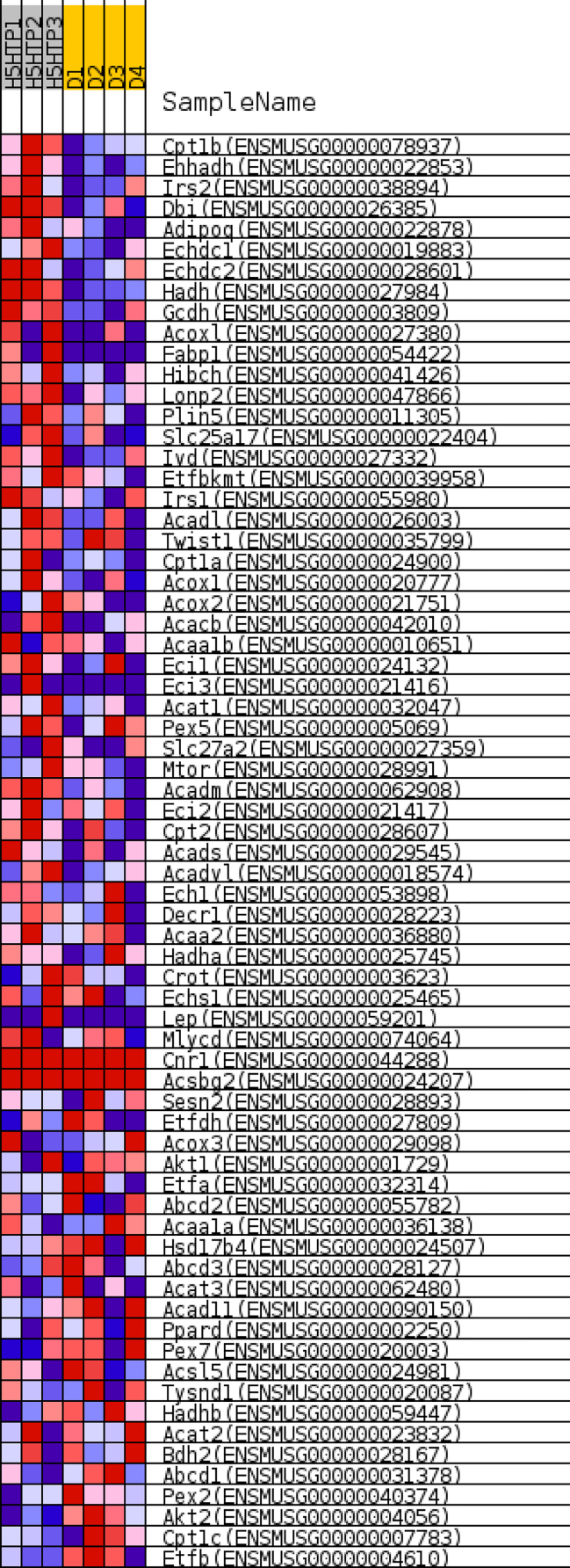


Suppl Figure4.


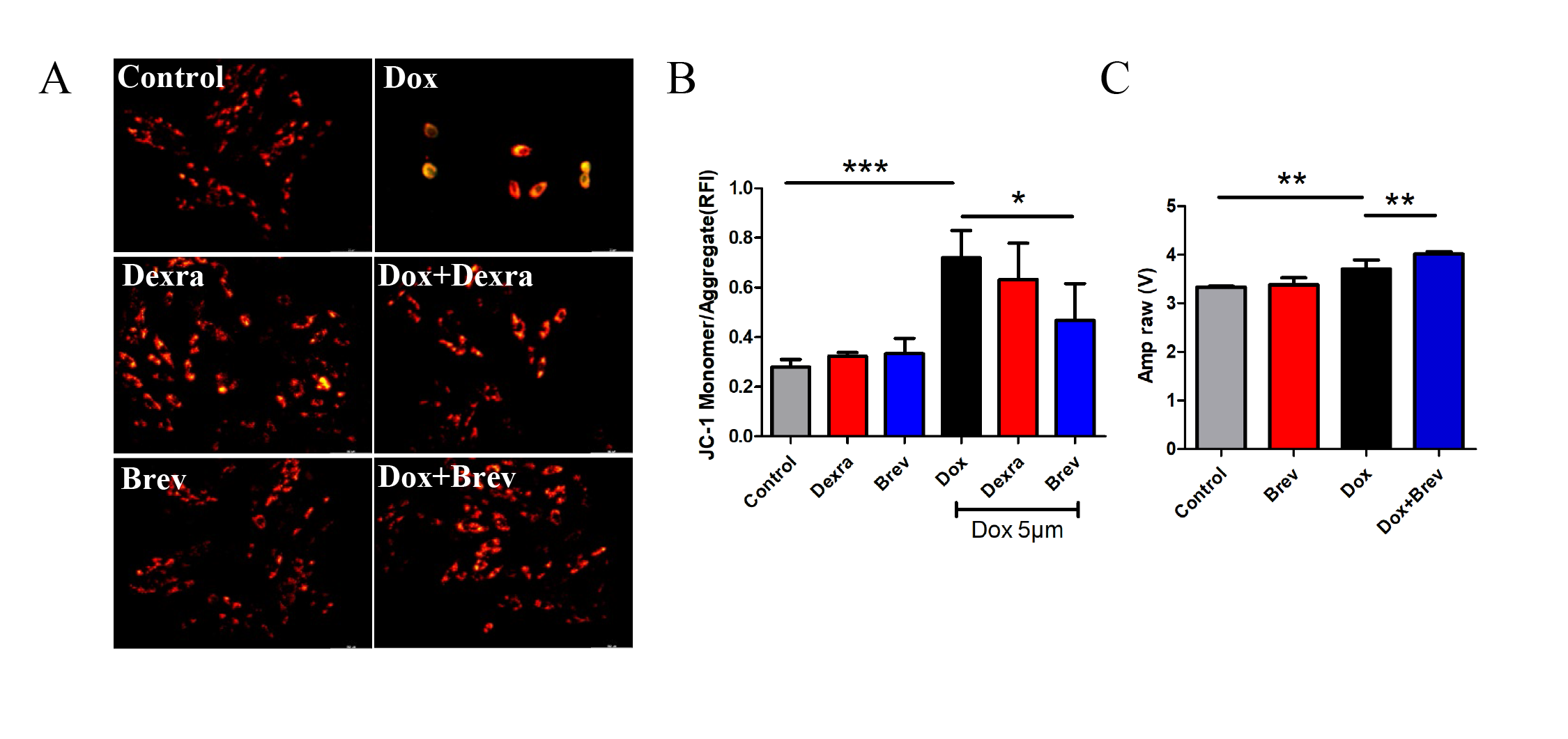


Suppl Figure5.


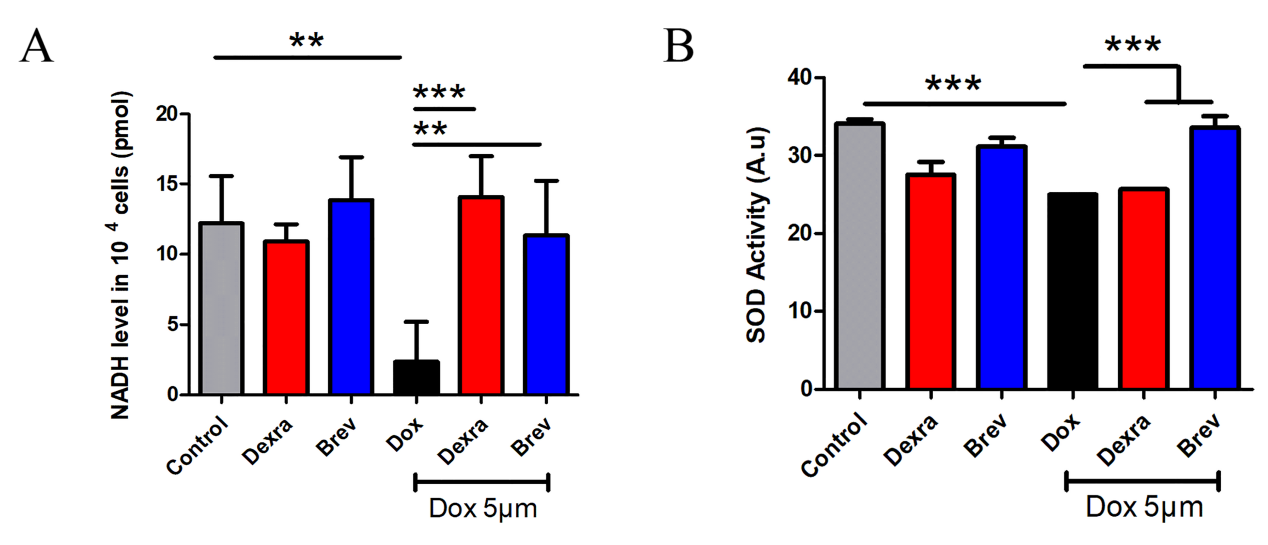


Suppl Figure6.


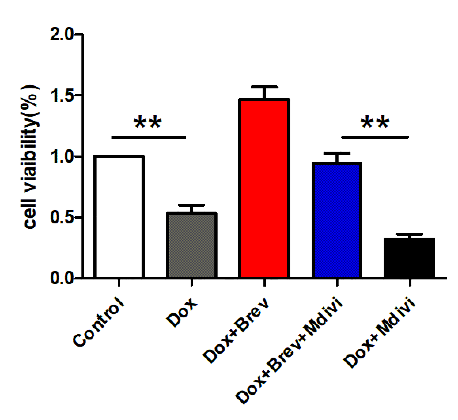


Suppl Figure7.


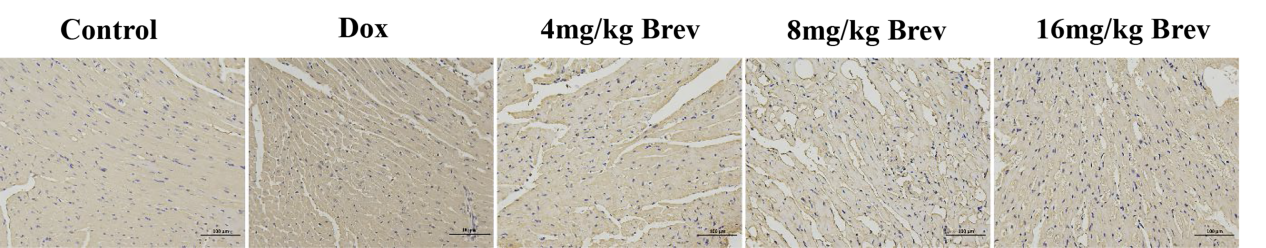

Supplement: Supplementary file 1 [file Table1.DOCX]
